# Use of traditional and complementary medicine for maternal health and wellbeing by African migrant women in Australia: a mixed method study

**DOI:** 10.1186/s12906-020-2852-6

**Published:** 2020-02-18

**Authors:** Zewdneh Shewamene, Tinashe Dune, Caroline A. Smith

**Affiliations:** 1 NICM Health Research Institute, Western Sydney University, Penrith, NSW 2751 Australia; 2 Center for Innovative Drug Development and Therapeutic Trials for Africa (CDT-Africa), Addis Ababa University, PO Box 9086, Addis Ababa, Ethiopia; 3 School of Health Sciences, Western Sydney University, Penrith, NSW 2751 Australia; 4 Translational Health Research Institute, Western Sydney University, Penrith, NSW 2751 Australia; 5 Graduate Research School, Western Sydney University, Penrith, NSW 2751 Australia

**Keywords:** Traditional medicine, Complementary and alternative medicine, Maternal wellbeing, African migrant women, Australia

## Abstract

**Background:**

Traditional medicine serves as a form of primary health care for more than 80% of African populations. Currently, there is no research documenting if and how African migrant communities engage with their traditional health practices and beliefs after they resettle in Western countries. The aim of this study was to examine African migrant women’s experiences and perspectives about traditional and complementary medicine use in relation to their maternal health and wellbeing in Australia.

**Methods:**

We conducted a mixed method study between December 2016 and October 2017. Questionnaires were completed by 319 women and 15 in-depth interviews were conducted among African migrant women residing across the Sydney metropolitan area, Australia. Survey data were analysed using SPSS (version 23) and logistic regression model was used to test associations. Qualitative data were analysed thematically using NVivo 11 software to identify themes and conceptual categories in the participants’ responses. The study was informed by Andersen’s Socio-behavioural model of health service utilisation.

**Results:**

The findings indicated that use of traditional and complementary medicine was high and continued to be well used following African women’s resettlement in Australia. The survey found that 232 (72.7%) women use some form of traditional and complementary medicine for maternal health and wellbeing purposes. Most women (179, 77.2%) reported that maintaining their maternal health and wellbeing was the most common reason for use. The interview findings indicated that access to traditional medicine included making requests from relatives and friends who travelled to Africa looking for a similar medicinal plant in Australia and preparing home remedies with advice from family members and healers back in Africa. Age ≥ 35 years (OR, 16.5; 95%CI, 6.58–41.5; *p* < 0.001), lower education (OR, 24; 95%CI, 8.18–71.1; *p* < 0.001), parity (OR, 7.3; 95%CI, 1.22–42.81; *p* = 0.029), and lower income (OR, 2.7; 95%CI, 1.23–5.83; *p* = 0.013) were strong predictors of traditional medicine use.

**Conclusion:**

Use of traditional and complementary medicine among African migrant women in Sydney remained high following resettlement in Australia. As noted in Andersen’s sociobehavioural model of health service utilisation, specific predisposing and enabling factors including age, education and income were associated with use of traditional and complementary medicine.

## Background

Australia has a growing and diverse African community [1]. The 2011 census data showed that there were close to 340,000 people of African descent living in Australia [1]. This number increased to around 390,000 African migrants in the 2016 Census. While the vast majority of Africans come to Australia as skilled migrants there is also a significant increase in arrivals from refugee countries such Ethiopia, Eritrea, Democratic Republic of Congo and Somalia [1]. In general, the state of New South Wales (NSW) has the largest number of permanent settler arrivals with 30% of the national total between 2006 and 2011 and approximately 40% of all humanitarian entrants [2, 3]. According to the 2016 census there were 86,410 African migrants.

Traditional medicine plays a crucial role for 70–90% of the African population as a primary health care option [4]. The practice of traditional medicine in Africa is a method of healing founded on its own concept of indigenous knowledge systems that developed over a long period of time within various societies [4]. Being practiced for countless generations well before the introduction of Western medical care, traditional medicine is culturally valued and accepted by most African communities [5–7]. Studies have shown that women in Africa extensively depend on cultural health practices for their maternal health and wellbeing [8, 9].

Australia has universal healthcare, including maternal services, with a public health insurance system, Medicare. Medicare provides treatment free of charge in public hospitals and subsidies the cost of out-of hospital treatment. The out-of-hospital treatment component of Medicare provides a rebate benefit for services, based on a proportion of a schedule of fees covering each type of service [10]. Although, conventional maternal healthcare is accessible in Australia, it is not clear if the widespread use of traditional medicine in Africa also continues following re-location in Australia among African migrant women [8]. As such, the present study examines the use of traditional and complementary medicine for maternal health and wellbeing among African migrant women in Australia. More specifically, the study seeks to determine the type of traditional and complementary medicine use, reasons and sources for use, and socio-demographic predictors of use of these therapies. Findings from this study will provide the opportunity to increase the knowledge and awareness of health professionals about the cultural health practices and beliefs of African migrant women in Australia. This will facilitate provision of culturally sensitive and responsive health services when caring for African migrant women by taking into account their cultural health practices and beliefs.

As defined by the World Health Organization [11], traditional medicine in this study refers to “the sum total of the knowledge, skills and practices based on the theories, beliefs and experiences indigenous to different cultures, whether explicable or not, used in the maintenance of health, as well as in the prevention, diagnosis, improvement or treatment of physical and mental illnesses”. Similarly, this study adopted the National Centre for Complementary and Integrative Medicine (NCCIM) definition for complementary and alternative medicine which states” *group of diverse medical and health care systems, practices, and products that are not generally considered part of conventional medicine”* [12].

## Theoretical framework

**Fig. 1:**
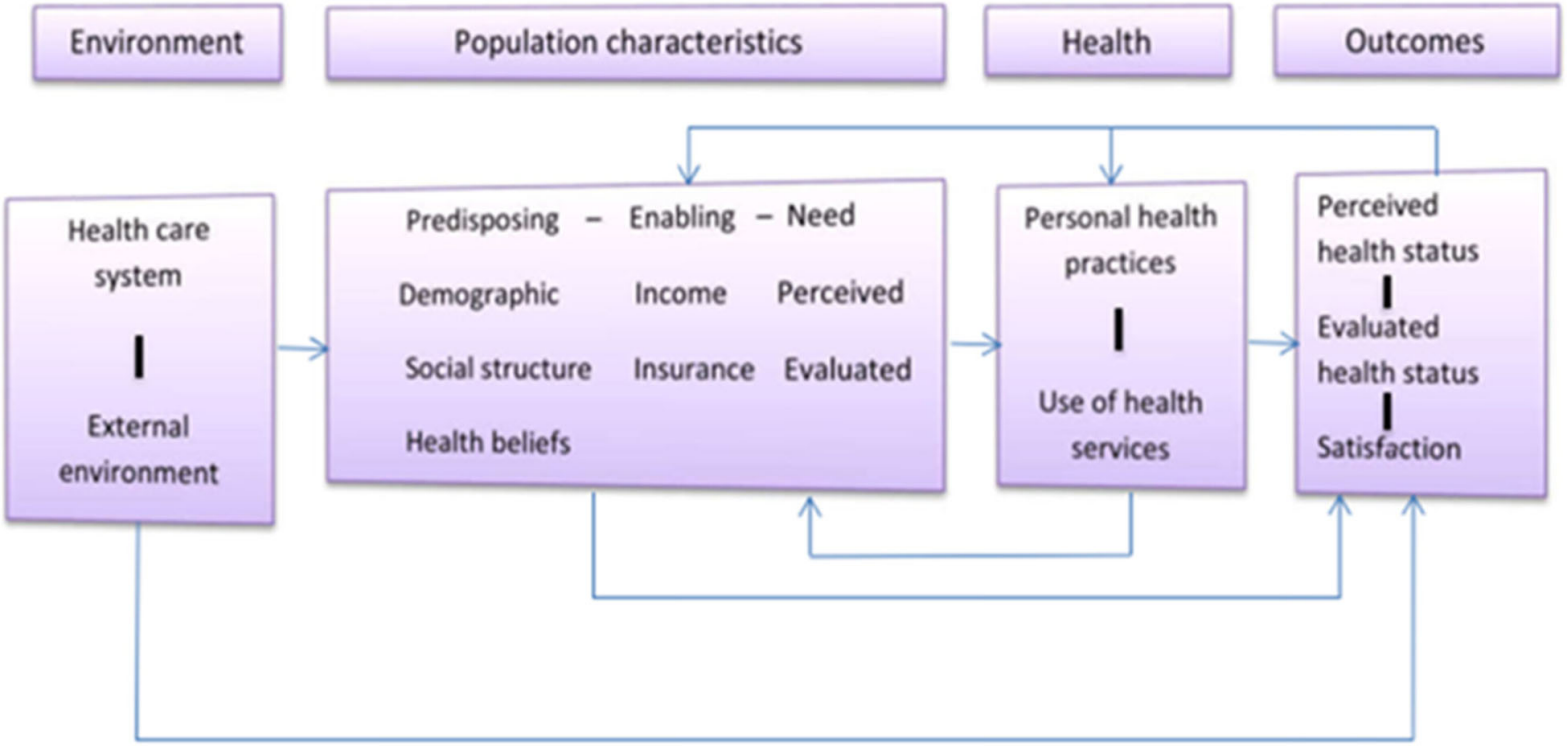
Core components of health seeking behaviour (Andersen, 1995, p. 8)

To understand factors that motivate the use of traditional and complementary medicine among African migrants, the Andersen’s sociobehavioural model of health service utilization was used [13]. It posits that health service utilisation depends on three core components which include: predisposing characteristics, enabling resources, and need factors. This theoretical framework also effectively integrates different factors which may influence the use of traditional and complementary medicine (Fig. 1). For instance, predisposing factors such as socio-demographic characteristics (e.g. age, gender, and education), knowledge, perceptions and attitudes, and race/ethnicity have been indicated to influence traditional and complementary medicine use [14–16]. Likewise, enabling factors such as income, insurance, access to and cost of conventional care have been shown to determine the use of traditional and complementary medicine [17, 18]. The need factors have also been consistently found to influence traditional and complementary medicine use based on previous studies [19, 20]. While human behaviors are complex, this model provides a comprehensive framework to describe health behaviors leading to utilization of traditional and complementary therapies.

## Methods

### Study design

A sequential explanatory mixed method study was undertaken from December 2016 to October 2017. Creswell and Creswell state that “explanatory sequential mixed methods is one in which the researcher first conducts quantitative research, analyzes the results and then builds on the results to explain them in more detail with qualitative research” [21]. This type of design is popular in fields with a strong quantitative orientation in which the initial quantitative results need to be explained further with the qualitative data [21]. In this study, data was collected sequentially with the survey results informing the development of the individual interview protocol. The survey examined the pattern of maternal traditional and complementary medicine use by African migrant women and the qualitative phase explored women’s experiences and perspectives about traditional and complementary medicine use in relation to their maternal health and wellbeing.

### Study participants

African migrant women residing across the Sydney metropolitan area were invited to participate in this study. Inclusion criteria were: born in Africa, aged ≥18 years, speak English, and had been a resident in Australia for at least 12 months. In addition, women were required to satisfy at least one of the following criteria: planning a pregnancy, attempted to conceive, have ever been pregnant, or had children. Women who were in Australia on short term work or holiday visas were excluded. In addition, women who nominated their willingness during the survey to participate in the interviews were purposively selected based on their experience of using traditional and complementary medicine for maternal health and wellbeing.

A verbal informed consent was obtained from all women who participated in the study. As the data collection involved online and telephone approaches, obtaining written consent was not possible. Ethical approval was received from Western Sydney University Human Research Ethics Committee (ref: H1196).

### Sampling strategy

A homogenous convenience sample of 319 Black African migrant women were included in the survey. According to Jager et al., (2017) one way to minimize the disadvantages of nonprobability sampling (lack of clearer generalisability) is through the strategic use of homogenous convenience samples (e.g. Black or White, male or females). Using this approach, the target population (not just the sample studied) was a specific sociodemographic subgroup.

When survey participants completed the questionnaires, they were invited to voluntarily nominate their willingness to be contacted for individual interviews. In addition to the inclusion criteria outlined under the survey, interview participants were selected purposively based on their experience of traditional and complementary medicine use for maternal health and wellbeing. Fifteen eligible women were interviewed. After 13 interviews, no new themes emerged which indicated saturation was achieved. In qualitative interviews, saturation occurs when no new or relevant data seems to emerge regarding the research objectives [22].

### Study promotion and recruitment procedures

The study was promoted through community consultations and networking with community associations, multicultural organisations, churches, and business firms which were affiliated with the African communities in Sydney. The study was advertised through posters and flyers displayed in prominent places including; shopping centers, train and bus stations, entertainment places, University and College campuses, restaurants, cafes, and African hair salons. For women expressing interest in the study a link to the online survey was sent through email, text messages and social media (Facebook, WhatsApp, and Viber). Optionally, paper-based questionnaires were hand delivered to individuals attending the organisations. Questionnaires were returned to the researchers using reply-paid and addressed envelopes.

### Data collection and measures

#### Surveys

Survey data were collected by using self-completed questionnaires (supplementary file 1). In addition, some participants completed an investigator administered questionnaires through face-to-face and over the phone [23]. Currently there are no standardised survey tools suitable to assess the use of traditional medicine among African migrant communities in Western countries. Therefore, the present study was based on a new survey tool developed by the authors from a comprehensive literature review. A researcher who develops a new measure should establish that it has “face validity” as a minimum requirement and that the new measure apparently reflects the content of the concept in question [24]. As an essentially intuitive process, the face validity of this study was ensured by using a mixed methods sequential explanatory design which allowed triangulation of quantitative and qualitative data on the same topic. The use of a mixed methods approach assured validating the survey findings through semi-structured in-depth interviews [25]. In addition, a pilot test performed before the main study ensured the face validity of the survey questionnaire.

The survey explored socio-demographic characteristics including age, marital status, pregnancy and birth, income, education and employment status, religion, and country of origin were collected using both open and closed questions. The survey also explored patterns of traditional and complementary medicine use in Australia relating to maternal health and wellbeing. Women were asked about the traditional and complementary health practices they used in Australia. A list of common traditional health practices was provided along with open-ended questions to facilitate participant recall. The survey also include question on specific maternal health related reasons for use of traditional medicine from preconception through to pregnancy and postnatal periods. A pilot test of the survey was undertaken to examine face validity. The pilot was carried out among eight African-born women. Following the pilot study, several modifications were made including revising terms to improve the readability of the questionnaire.

#### Interviews

The development of the interview guide was informed by the preliminary analysis of the survey data to women’s experience of using traditional and complementary medicine for maternal health and wellbeing. Data for the interviews was collected using a digital audio-recorder and notes were taken by the researcher during and immediately following the interview. Interviews lasted between 40 and 80 min. All audio-recorded interviews were transcribed in full. The first interview was transcribed by the researcher and subsequent audio-recorded interviews were transcribed verbatim by a transcription company (https://www.pacifictranscription.com.au/). The interview explored in more detail women’s experiences and perceptions of using traditional medicine during preconception, pregnancy, and/or postnatal periods (supplementary file 2). Interview results were presented by way of pseudonyms.

#### Data analysis

Survey data was checked for consistency, coded appropriately, and entered into SPSS software package version 23. Descriptive analysis using frequencies and proportions was conducted to describe the sample characteristics and distribution of variables. The relationship between traditional medicine use and independent factors was examined using binary logistic regression model. For all significance tests a *p*-value of 0.05 or less and a 95% confidence interval were used. Bivariate logistic analysis was initially conducted with potential predictor variables to establish their association with use of traditional medicine. Following the bivariate analysis, multivariate logistic regression analysis was conducted to control for possible confounding variables and to compute the adjusted odds ratios (OR) for the explanatory variable. All variables with a *p*-value of less or equal to 0.25 were selected and entered simultaneously into a logistic regression model because the traditional level of 0.05 can fail to identify important variables [26]. A stepwise backward elimination method was employed to eventually produce the most parsimonious model. In backward elimination method, variables with greater p-value were sequentially eliminated until all variables in the model were *p* < 0.05 [27].

Qualitative data were analysed thematically using NVivo 11 software program [28] to identify themes, conceptual categories, commonalities and differences in the participants’ responses. The analysis was started by listening to the recoded audio and reading and re-reading the transcripts several times [29]. This process of familiarisation or immersion into the data helped the researcher to reflect on the entirety of the data and better understand and interpret the women’s views about traditional medicine. The next step was generating codes and using NVivo 11 to produce a concise matrix of key emerging ideas. Finally, a coding summary and report was produced with the sub-themes that make meaningful contributions to answering the research questions. The first author conducted the data analysis and generated the summary codes. The second and third authors reviewed the interview transcripts, the coding summary and provided feedback for revisions to coding framework which were then incorporated into the qualitative data analysis by the first author. Names have been changed to protect the identity of women.

## Results

A total 634 survey questionnaires were distributed and 348 (54.9%) surveys were returned (205 online and 143 paper-based). Twenty-nine questionnaires (25 online and 4 paper-based) had significant missing data due to unanswered questions and were disregarded. A total of 319 completed survey questionnaires were analysed.

A sub-sample of 15 women who nominated their interest during the survey completed one-to-one interviews. Eleven interviews took place over the phone and four were undertaken face-to-face. After 13 interviews, no new themes emerged which indicated that saturation was achieved.

### Socio-demographics of participants

Forty-three nations in Africa were represented in this study. One hundred and thirty-one women (41.1%) were aged between 25 and 34 years (Table 1). Two hundred and forty two (75.8%) of the participants were currently employed. The majority of women (208, 65.2%) reported annual household income of less than 50,000 (AUD$). Many of the women were married (203, 63.6%). Overall, 235 (73.7%) and 225 (70.5%) participants had been pregnant at least once and had at least one child respectively.

**Table 1:** Demographic characteristics of survey participants

Characteristics		N (%)
Age	18–24 years	25 (7.8)
	25–34 years	131 (41.1)
	35–44 years	115 (36.1)
	45–54 years	34 (10.7)
	55 or older	14 (4.1)
Annual household income (AUS$)	0–24,000	96 (30.1)
	25,000-49,999	112 (35.1)
	50.000–74,999	65 (20.4)
	75,000-99,999	25 (7.8)
	100,000 or above	21 (6.6)
Marital status	Married or living together	203 (63.6)
	Single	82 (25.7)
	Divorce/Separated/widowed	34 (10.7)
Gravidity	Never been pregnant	84 (26.3)
	Has been pregnant at least once	235 (73.7)
Parity	No children	94 (29.5)
	Have at least one child	225 (70.5)
Education status	Less than high school	6 (1.9)
	High School graduate	33 (10.3)
	Certificate or diploma level	127 (39.8)
	University degree	117 (36.7)
	Postgraduate degree	36 (11.3)
Employment status	Unemployed	27 (8.5)
	Full time	99 (31)
	part time	143 (44.8)
	self employed	25 (7.9)
	other	25 (7.8)
Religion	African Traditional	14 (4.4)
	Christian	203 (63.6)
	Muslim	97 (30.4)
	Others	5 (1.6)

Women in the interview also had diverse backgrounds from different regions in Africa, such as Nigeria, Cameroon, and Sierra Leone, in West Africa; Ethiopia, and Tanzania in East Africa; Rwanda and Gabon in Central Africa; Sudan in Northern Africa; and Zimbabwe in Southern Africa (Table 2). The majority of participants had arrived to Australia on Family Visas.

**Table 2:** Characteristics of interview participants

S. no	Name	Education	Age at arrival in Australia	Length of time in Australia, in year	Number of children	Religion	Visa type on entry	English proficiency (Self rated)
1	Fiona	Secondary school	38	21	3	Christian	Humanitarian /Refugee	Good
2	Zena	University degree	30	32	3	Catholic	Humanitarian /Refugee	Excellent
3	Soy	Postgraduate degree	38	8	3	African traditional	Skilled/work	Good
4	Sim	University degree	22	11	2	Christian	Family/partner	Good
5	Anna	University degree	32	10	1	Christian	Humanitarian /Refugee	Excellent
6	Tia	Postgraduate degree	26	2	2	Christian	Student	Good
7	Mary	Diploma	18	30	4	Christian	Humanitarian /Refugee	Excellent
8	Eva	University degree	26	2	1	Christian	Student	Fluent
9	Saly	University degree	26	22	2	Christian	Family/partner	Excellent
10	Hiva	University degree	19	11	1	Christian	Family/partner	Excellent
11	Ney	Postgraduate degree	10	20	0	Christian	Family/partner	Fluent
12	Mire	University degree	23	2	0	Christian	Skilled/work	Good
13	Lita	Certificate	32	3	2	Christian	Family/partner	Excellent
14	Jay	Postgraduate degree	10	16	0	Christian	Student	Good
15	Ema	University degree	20	5	2	Muslim	Family/partner	Good

### Patterns of traditional and complementary medicine use

Use of traditional or complementary medicines was high with 232 (72.7%) women using some form of traditional or complementary medicine (Table 3). The most frequently used traditional or complementary medicine products and services were herbal medicine (163, 61.7%), prayer for health (146, 55.3%), vitamins (88, 33.3%), massage (87, 33%), and faith healer services (81, 30.9%).

**Table 3:** Common forms of traditional and complementary medicines used by African migrant women

Types of traditional or complementary medicine used	n (%)
Any type of complementary or traditional medicine	232 (72.7)
Herbal medicine	163 (61.7)
Prayer for health	146 (55.3)
Vitamins	88 (33.3)
Massage	87 (33.0)
Faith healer	81 (30.9)
Traditional Chinese Medicine	72 (27.3)
Chiropractic	30 (11.4)
minerals	25 (9.5)
Antioxidants	21 (8.0)
Yoga	18 (6.8)
Meditation	23 (8.7)
Diet therapy	19 (7.2%)

### Reasons for use of traditional or complementary medicine

**Fig. 2:**
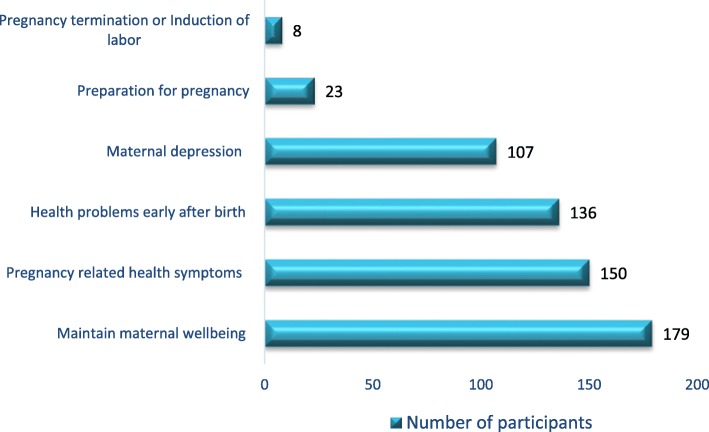
Maternal health related reasons for use of traditional and complementary medicine among African migrant women

The majority of women identified maintaining their wellbeing was the most common reason for using traditional or complementary medicine prior to conception, during pregnancy and the postnatal period (179, 77.2%). The other common reasons reported by the participants were pregnancy related symptoms (150, 64.7%) (Fig. 2).

Interview participants confirmed these survey findings. In one interview, for example, Ema commented, *“I have used various traditional medicines, depending on the circumstance. Not necessarily because I am sick, but also as a prevention strategy and for wellbeing.”* For some participants, using these practices was perceived as a balancing mechanism to the modern-day lifestyle in which more unhealthy foods were consumed with less physical exercise.

The modern lifestyle here is like no exercise, junk foods and all artificial products. So I am more conscious with which health choice I should make this time. My African knowledge about this traditional medicine helps me a lot (Ema)*.*

Another reason for using traditional and complementary medicine related to specific maternal health conditions*.* For instance, many participants indicated that they used different forms of traditional medicine to prevent and treat nausea and vomiting in the first trimester of their pregnancy. According to Tia,

I normally used homemade remedies like I put some honey with my tea; and especially when I was pregnant I was using lemon with water, because during first trimester pregnancy, you know, there is nausea and vomiting (Tia)*.*

Ema also mentioned, *“I used herbal stuffs during my pregnancy because I have sort of nausea and vomiting.”* The use of traditional and complementary medicine was also indicated for losing weight during pregnancy. Hiva, a mother of two stated, *“I do use herbal things like herbal tea. Herbal tea and also other herbal products that make you lose weight during pregnancy.”*

During the postnatal period, use of traditional medicine was reported for a number of specific reasons. For instance, respondents indicated that traditional medicine was believed to restore a woman’s pre-pregnancy body shape and strength after giving birth.

It's well known that when a woman has a baby, she needs to go through this process [hot water bath using herbal fragrances] to get back her body in the sense of mobility, flexibility, prevent the soreness and disease (Soy).

According to Sim, *“soon after giving birth, it [traditional medicine] was believed it would help me to get stronger and be able to get back to normal shape.”* Ema explained, “*after giving birth I was not strong for a long time and that’s why I was depending on traditional medicine.”* Women also reported that they used herbal medicine for increasing breast milk production. For example, Ema said, *“that food cooked with herbs was good for me to produce more milk.”*

Some participants reported that they used traditional and complementary medicine to help them prepare for pregnancy or to enhance the chance of conception.

Even before my pregnancy I used these herbs, but I don't remember the name, but my mum recommends me to increase the chance of fertility. Because I have a delayed pregnancy for three or four years after marriage. So I always took herbs with food that my mother cooks. She also has some special foods mixed with different herbs and spices that she gives me to become pregnant (Ema)*.*

### Sources of information about traditional medicine

To provide more insight into who provided the information and how it influenced use of traditional and complementary medicine, the participants were asked during the interviews about accessing their culture specific traditional therapies and the process of knowledge acquisition related to traditional medicine in their respective cultures. In response, many women discussed that the information or knowledge about traditional medicine was handed down through their families to maintain good health and to treat illnesses.

For example, Ney stated that her main source of knowledge about traditional medicine was from her close family members.

I grew up with somebody [her mother] who is very much who looks outside of western medicine. Through my mum's influence I always seek alternative ways of looking after my health. I've always sought to use certain herbal soups, or probiotics (Ney)*.*

The knowledge or information about use of traditional medicine for maternal health purposes was also learnt from mothers or grandmothers. In her comment on how she learned about traditional medicine from her mother, Sim remarked:

It's like, when I got pregnant, when I gave birth, my mum told me, oh this will help you to get this or that- so I think that's how it gets passed on. She probably got it from her mum and her mum got it from her mum. So, I'd probably say the same thing to my daughter. I think it was passed on This is how I had traditional medicine and it helped me. So probably maybe generation to generation I think (Sim)*.*

For some participants, traditional medicine was accepted because of their families prior practice or use. The most important thing in this case was not the effectiveness, but rather the belief and trust the family held towards traditional medicine.

I just have, you know, there are times we say traditional medicines are just believing. So I have been used to drinking this bitter root they call bamba in my language, so we've been used to drinking it in the morning. My mother used to soak it. Sometimes she boils it and then give it to us. I don't really go, we don't really go to any other traditional healers or whatever it is. We just believe in drinking that thing that my mother gives me (Fiona)*.*

Anna recalled how she was excited when her husband told her that the traditional medicine which she used to use it in Africa was also available in Australia*. “So when I came here, it was just - when my husband told me about he found this tree here, it was just a continuation. I just drink.” Some p*articipants also reported that their African friends were an important source of information about traditional or complementary medicine in Australia.

I have a lot of friends who are just very much in this alternative way of living, sort of modern day hippies, and then somebody said, oh try this, try this. It's your root so why don't you just try and release whatever it is that you're hanging on. Look, I don't know if it worked or not. I don't know if it was the placebo, but I just remember that I took her advice and I tried different traditional medicine (Ney)*.*

I can get the information from my friends here, from relatives, even sometimes I call my mum back home to tell me what to use for such and such problems and she will teach me how to prepare that [traditional medicine] (Ema)*.*

A participant indicated that she accesses specific herbal products sent directly from Africa or through sharing from friends and relatives.

Well I have a habit of asking my mother to send me some plant medicine powder that she got from local healers. Whenever, some friends go back to Africa for a holiday, I will ask them to bring that for me. Sometimes I also ask relatives and friends to share with me if they have one, usually these African herbal medicines good for many illnesses but I don’t remember the name of the plant (Mire)*.*

### Socio-demographic predictors of traditional and complementary medicine use

A multivariate logistic regression analysis was undertaken to determine the predictive factors of traditional and complementary medicine use (Table 4). Older women (≥ 35 years) were 16.5 times more likely to use these practices for maternal wellbeing than women aged < 35 years (*p* < 0.001). Women with lower income and educational level were 2.7 and 24.0 times more likely to use traditional and complementary medicine for their maternal wellbeing compared to those with higher income (*p* = 0.013) and educational status (*p* < 0.001) respectively. Primiparous women were 7.3 times more likely to use these practices for maternal wellbeing compared to nulliparous women (*p* = 0.029).

**Table 4:** Sociodemographic predictors of traditional and complementary medicine use for maternal wellbeing among African migrant women after resettlement in Australia

Factors		Total (*n* = 319)	Use of traditional and complementary medicine for maternal wellbeing		OR	95%CI	*P*-value
			Yes (*n* = 232)	No (*n* = 87)			
Age	< 35 years	156 (48.9%)	77 (33.2%)	79 (90.8%)	-	-	-
	≥ 35 years	163 (51.1%)	155 (68.8%)	8 (9.2%)	16.5	6.58–41.51	< 0.001
Household income (AUS$)	< 50,000	208 (65.2%)	175 (75.4%)	33 (37.9%)	2.7	1.23–5.83	0.013
	≥50,000	111 (34.8%)	57 (24.6%)	54 (62.1%)	-	-	-
Marital status	Single or separated	116 (36.4%)	71(30.6%)	45(51.7%)	-	-	-
	Married	203 (63.6%)	161 (69.4)	42 (48.3%)			
Gravidity	Never been pregnant	84 (26.3%)	47 (20.3%)	37 (42.5%)	-	-	-
	Has been pregnant at least once	235 (73.7%)	185 (79.7%)	50 (57.5%)			
Parity	No children	94 (29.5%)	49 (21.1%)	45 (51.7%)	1	-	-
	Have at least one child	225 (70.5%)	183 (78.9%)	42 (48.3%)	7.3	1.22–42.81	0.029
Education status	Certificate or below	166 (52%)	161 (69.4%)	5 (5.7%)	24.1	8.18–71.1	< 0.001
	Diploma or above	153 (48%)	71 (30.6%)	82 (94.3%)	-	-	-

## Discussion

Currently, research on African migrants’ traditional health practices and how this may influence their health-seeking behaviour in Western countries is limited. This is the first mixed methods study which generated a comprehensive understanding of the cultural health practices and beliefs of African migrant women in Australia. Triangulation largely resulted in consistency across the survey and interview findings and increased the trustworthiness and validity of the conclusion form this study.

The findings indicated that use of traditional and complementary medicine among African migrant women was high and continued use following migration. Being practiced for countless generations even well before the introduction of Western medical care, traditional medicine is highly valued and culturally accepted by most African communities [5–7]. As such, the findings in this study might be a reflection that African migrant women in Australia continue their cultural health practices because of their health and illness beliefs, cultural acceptance, and concept of holistic health related to traditional medicine.

This study’s findings demonstrated that herbal medicine was the most common form of traditional medicine used by African migrant women. Respondents indicated that the knowledge and experience of gathering and preparation of herbal medicines was a common practice in their families in Africa. The result of this study may suggest the pre-migration experience of African women played a significant role in continuing use of herbal medicine in Australia.

Our study found that spiritual healing (prayer for health purposes with or without consulting faith healers) were commonly utilised by African migrant women. Previous studies also identified that the practice of prayer for health was common among migrant African communities [30–33]. Brown et al., (2007) found that prayer was the most common traditional health practice among African Americans compared to other minority groups [33]. A study reviewing the influence of spiritual beliefs and practices on the treatment preferences of African Americans indicated that spirituality is an important part of African-American culture and spiritual beliefs are important in understanding and coping with illness and provided a framework within which treatment decisions are made [31]. In another study, African Americans held beliefs that consider prayer as the most important part of their health care and influences their medication decisions [34]. In addition, African Americans were more likely to believe that the power of spirituality and prayer promotes healing and treatment of active health complaints than other minority groups [35–39]. Similarly, the present study findings have shown that prayer was used not only for religious purposes but also as part of maintaining the general wellbeing and treatment of specific maternal health complaints.

This study also found that that African migrant women report the use of complementary therapies which were not common in Africa but are widely used in the Australian population. Use of these modalities included vitamin/minerals, massage, and chiropractic, and traditional Chinese medicine. As such, the present finding may suggest that African migrant women are open to explore and experience complementary therapies outside of their culturally specific health practices. Perhaps, this might also be an attempt to substitute their cultural health practices that they were unable to easily access in Australia. Study findings indicated that services from traditional birth attendants, bone setters, and diviners were common practices women utilised while they were in Africa but least mentioned after their resettlement to Australia. On the other hand, this finding may indicate that African migrant women also hold similar views with Australia complementary medicine users. For example, some of these views including the perception of complementary medicine as holistic, preventive, safe and that complementary medicine practitioners are more supportive towards their health compared to other health professionals were also identified among the general Australian population [40].

Many women in the study identified that maintenance of their maternal wellbeing was the most common reason for use of traditional and complementary medicine. This study also found that treatment of specific maternal health complaints during antenatal and postnatal periods were also a common reason for use of traditional medicine. Previous studies also revealed that African women who have health complaints during pregnancy or after birth were more proactive about their health and were accessing a broader range of traditional and complementary therapies [8, 41]. This use could start from the preparation phase for pregnancy through to the antenatal and postnatal periods. Some women reported that the use of traditional and complementary medicine was associated with the perception that it increases immunity. Similarly, traditional medicine was often perceived as a better preventive strategy and regarded as more natural, safe, and/or having at least equal efficacy when compared with medical prescriptions for maternal wellbeing among African women in Africa [42, 43]. As such, the present finding may suggest that previous perceptions among African migrant women were important to continued us of traditional medicine because they believed that it has a medicinal value which was important to protect their general wellbeing.

The present study identified that African migrant women use various approaches to access their cultural traditional medicines in Australia. These sources included sharing from relatives and friends who recently traveled to Africa and brought back some traditional medicines, looking for a similar medicinal plant in Australia, and preparing home remedies with advices from family members and healers back in Africa. This finding was similar to research with other migrant groups which highlighted that migrant communities may depend on family members and traditional healers who came from their own country of origin to access their cultural health practices. Mexican migrants in US rely heavily on transnational networks to meet their health care needs [44]. According to the study, through their close family recommendations, Mexican migrants were able to address their health care needs in a way that is culturally appropriate to them by involving family, friends, other migrants, parcel services, long distance calling booths, and ethnic shops to access culture specific traditional medicine. However, participants in the present study identified that across many African migrant communities in Australia there were no traditional practitioners (except faith healers) who were themselves African migrants.

As noted in the Anderson’s sociobehaviour model of health service utilisation, specific predisposing and enabling factors including age, education and income were associated with use of traditional and complementary medicine. Our analysis found that lower educational level was significantly correlated with increased traditional and complementary medicine use for maternal health and wellbeing. Similar to this finding, other studies have also reported that African women who use traditional medicine were more likely to be less educated [45, 46]. Increased use of traditional medicine among the less educated group in this study could be partly explained by the fact that lower education may limit African women’s knowledge about available healthcare options in Australia. Additionally, African migrant women with less educational level may also have lower health literacy about the Western medical care and thus embrace their traditional beliefs and practices as first line of maternal health care in Australia.

Low income was also strongly associated with use of traditional medicine for maternal health and wellbeing among the African migrant women. Previous study findings indicated that women with low income may experience limited autonomy as compared to their male counterparts (on whom they may be dependent) who may be responsible for making decisions concerning women’s health care choices and wellbeing [47, 48]. Our analysis also shows that older age (≥ 35 years) and having children were associated with an increased use of traditional medicine for maternal wellbeing and is consistent with previous studies [41, 49–51]. This could be possibly due to the fact that these women may have spent more time in Africa and thus had pre-migration experiences of using traditional medicine.

### Implications

The findings from this study can be used to increase the knowledge of healthcare professionals about the African migrant women’s cultural health practices. This in turn will facilitate provision of culturally sensitive and responsive health services when caring for African migrant women by taking into account their cultural health practices. Further research exploring specific traditional and complementary medicine modalities such as herbal medicines will have implications for users and health professionals with respect to information on safety, effectiveness, and appropriate utilisation of these products. Studies examining health beliefs of African migrant women and how traditional health practices and beliefs may influence utilisation of Western medical care are needed.

### Strengths and limitations

There are many key strengths inherent in this study. This study which generated a comprehensive understanding of the cultural health practices and beliefs of African migrant women in Sydney. As such it filled an important knowledge gap in the literature. A key methodological strength of the study was the use of a mixed methods approach in bringing together the different strengths of the quantitative and qualitative methods and then validating the survey findings through semi-structured in-depth interviews through data triangulation.

A key limitation was the use of nonprobability sampling strategy which may affect generalisability of the findings. Random sampling was not possible for this study because of the unknown sampling frame of African migrant women population who can meet the selection criteria. However, efforts were made to try and approximate random sampling by eliminating as many biases as possible. Some of such efforts were: 1) the use of homogenous convenience sampling strategy instead of conventional convenience sampling, 2) a fairly large sample for a relatively small population size of African migrant women in Australia, and 3) recruitment of participants across multiple venues including community organisations, market places, religious institutions, colleges and Universities, entertainment places, event and gathering sites, and train and bus stations. In addition, taking the sample only from the geographic area of the Sydney metropolitan area may also limit the generalisability of the findings.

## Conclusion

In summary, our finding indicated that use of traditional and complementary medicine is high among the African migrant women. African migrant women use traditional and complementary medicine for health maintenance and treatments of specific health conditions at various points from preconception through to the postnatal period.

## Supplementary information

**Additional file 1.** Survey Tool.

**Additional file 2.** Interview guide.

## Data Availability

The supporting materials used in this study are contained within the article.

## Abbreviations

CAM: Complementary and alternative medicine
NICM: National Institute of Complementary Medicine
SPSS: Statistical package for social sciences
WHO: World Health Organization
