# Supplementary material for: Use of traditional and complementary medicine for maternal health and wellbeing by African migrant women in Australia: a mixed method study
Source: BMC Complement Med Ther. 2020 Feb 18;20:60. doi: 10.1186/s12906-020-2852-6 (PMC7076811; doi:10.1186/s12906-020-2852-6)
Supplement: Supplementary file 1 — Additional file 1. Survey Tool. [file 12906_2020_2852_MOESM1_ESM.pdf]

Supplementary file 1: Survey Tool

Section one: Use of traditional and complementary medicine

1. Have you ever used any sort of traditional or complementary medicine in **Africa**? (Please encircle all that apply)
    1. Herbal medicine
    2. Prayer for health
    3. Traditional birth attendant/traditional midwife
    4. Bone settlers/traditional osteopath
    5. Diviners/witchcrafts
    6. Faith healers/spiritual healers
    7. Aromatherapy
    8. Traditional Chinese Medicine (TCM)
    9. Vitamins
    10. Minerals (e.g. Zinc, selenium)
    11. Antioxidants
    12. Yoga
    13. Ayurveda
    14. Biofeedback
    15. Osteopathy
    16. Energy healing
    17. Chiropractic
    18. Massage
    19. Tai chi/qi gong
    20. Guided imagery
    21. Progressive relaxation
    22. Deep breathing
    23. Meditation
    24. Diet-based therapies (eg, vegetarian)
    25. Other: specify ____
    26. No, I haven't used any of them (**go to question 4**)
2. If you have used at least one traditional/complementary medicine in **Africa**, why did you choose to use it? (Please encircle all that apply)
    - A. Traditional/complementary medicine is cheaper than western medicine in Africa
    - B. Western medicine is not accessible in Africa
    - C. It is more safer
    - D. It is more effective
    - E. It is natural
    - F. Some health problems can only be treated by traditional/complementary medicine
    - G. Traditional/complementary medicine is more holistic (eg. It combines body and mind)
    - H. Traditional/complementary medicine is more valued in my culture/community
    - I. Other reason: specify ____

3. Have you ever used any traditional and complementary medicine for the following maternal health conditions in **Africa**? (Please encircle all that apply)
   A. Preparing for pregnancy (e.g., fertility and preconception)
   B. Any pregnancy related health problems (nausea, vomiting, back pain, leg cramps, sleeping problems, cravings, hypertension, diabetes, bleeding, urinary tract infection)
   C. Pregnancy termination/induction of labour
   D. Maintain wellbeing during and after pregnancy
   E. Early postnatal period (e.g., to improve milk section)
   F. Maternal depression
   G. Other purpose, specify____
   H. I don't use them for maternal health purposes
4. Have you ever used any sort of the following traditional or complementary medicines in **Australia**? (Please encircle all that apply)
   1. Herbal medicine
   2. Prayer for health
   3. Traditional birth attendant/traditional midwife
   4. Bone settlers/traditional osteopath
   5. Diviners/witchcrafts
   6. Faith healers/spiritual healers
   7. Aromatherapy
   8. Traditional Chinese Medicine (TCM) (eg. Herbs, acupuncture)
   9. Vitamins
   10. Minerals (e.g. Zinc, selenium)
   11. Antioxidants
   12. Yoga
   13. Ayurveda
   14. Biofeedback
   15. Osteopathy
   16. Energy healing
   17. Chiropractic
   18. Massage
   19. Tai chi/qi gong
   20. Guided imagery
   21. Progressive relaxation
   22. Deep breathing
   23. Meditation
   24. Diet-based therapies (eg, vegetarian)
   25. Other: specify ____
   26. No, I haven't used any of them (**go to questions 7**)
5. If you have used at least one traditional/complementary medicine in **Australia**, why did you choose to use it? (Please encircle all that apply)
   A. Traditional/complementary medicine is cheaper than western medicine in Australia
   B. To support the treatment outcome from western medicine
   C. It is more safe
   D. It is more effective
   E. It is natural
   F. Some health problems can only be treated by traditional/complementary medicine
   G. Traditional/complementary medicine is more holistic (eg. It combines body and mind)
   H. Traditional/complementary medicine is more valued in my culture/community
   I. Other reason: specify ____
6. Have you ever used any traditional and complementary medicine for the following maternal health conditions in **Australia**? Choose all that apply
   A. Preparing for pregnancy (e.g., fertility and preconception)
   B. Any pregnancy related health problems (nausea, vomiting, back pain, leg cramps, sleeping problems, cravings, hypertension, diabetes, bleeding, urinary tract infection)
   C. Pregnancy termination/induction of labour

D. Maintain wellbeing during and after pregnancy
E. Early postnatal period (e.g., to improve milk section)
F. Maternal depression
G. Other purpose, specify____
H. I don't use them for maternal health purposes

7. How did you get information about traditional and complementary medicine in **Africa**?
   A. Family and friends
   B. The media
   C. Internet/social media
   D. General practitioners
   E. Obstetricians/Midwives
   F. Traditional/faith healers
   G. Traditional birth attendant
   H. Doula
   I. Other sources: specify ____
8. How did you get information about traditional and complementary medicine in **Australia**?
   A. Family and friends
   B. The media
   C. Internet/social media
   D. General practitioners
   E. Obstetricians/Midwives
   F. Traditional/faith healers
   G. Traditional birth attendant
   H. Doula
   I. Other sources: specify ____

Section two: attitudes towards traditional and complementary medicine

9. Please indicate the extent that you agree or disagree with the following statement. (encircle 1 if strongly agree ……. 5 if you strongly disagree)

Statements	Strongly agree	Agree	Neutral	Disagree	Strongly disagree
Traditional and complementary medicine boosts my immune system/resistance	1	2	3	4	5
Traditional and complementary medicine promotes a holistic approach to health	1	2	3	4	5
Traditional and complementary medicine gives me more control over my health/body	1	2	3	4	5
Traditional and complementary medicine is cheaper than conventional medicine	1	2	3	4	5
Traditional and complementary medicine is a better preventative measure than conventional medicine	1	2	3	4	5
It is easier to find Traditional and complementary medicine than having prescription drugs	1	2	3	4	5

Traditional and complementary medicine is more effective than conventional medicine	1	2	3	4	5
Traditional and complementary medicine is natural and has fewer side-effects than conventional medicine	1	2	3	4	5
Evidence of effectiveness is important to my choice of Traditional and complementary medicine	1	2	3	4	5
My personal experience of the effectiveness of Traditional and complementary medicine is more important than clinical evidence	1	2	3	4	5
Traditional and complementary medicine needs to be tested for safety/side-effects	1	2	3	4	5
Traditional and complementary medicine practitioners listen to and spend more time with their patients than doctors	1	2	3	4	5
Midwives should be able to advise their patients about commonly used Traditional and complementary medicine	1	2	3	4	5
General practitioners should be able to advise their patients about commonly used Traditional and complementary medicine	1	2	3	4	5
Obstetricians/gyneacologists should be able to advise their patients about commonly used Traditional and complementary medicine	1	2	3	4	5

Section three: perceived health status and health seeking behaviour

10. In general would you say your health is:
    A. Excellent
    B. Very good
    C. Good
    D. Fair
    E. Poor

11. In Australia, have you ever experienced any of the following maternal health problems? (Please select all that apply)
    A. Unable to conceive/fertilization problem
    B. Pregnancy related health problems (e.g. nausea, vomiting, leg ramps, virginal bleeding, anemia, urinary tract infection, back pain, cravings, diabetes, hypertension, tiredness etc.)
    C. Miscarriage/failed pregnancy
    D. Lack of milk secretion
    E. Maternal depression
    F. Others: specify____
    G. I did not experience such problems
12. Please rate how commonly you consulted/used the following people/services for any women's health issues in Australia (encircle 1 if you never used ……. 5 if you frequent used)

People/services	Never	Rarely	sometimes	frequently

GP	1	2	3	4
Specialist	1	2	3	4
Midwives/Nurses	1	2	3	4
Traditional healer/practitioner	1	2	3	4
Self-treatment with traditional/complementary therapies	1	2	3	4
Self-treatment with over-the-counter medications	1	2	3	4

Section four: barriers in accessing conventional health care

13. Are you entitled to access medical care through Medicare?
    A. Yes
    B. No
14. Does your health insurance cover pregnancy-related care
    A. Yes
    B. No
15. Do you feel that you experience discrimination because of your race, ethnicity, colour, or country of origin when seeking medical care?
    A. Often
    B. Sometimes
    C. Rarely
    D. Never
    E. Not sure
16. In the past 12 months; was there a time when you needed medical care, but did not seek treatment?
    A. Yes
    B. No
    If yes, what was the main reason ____
    ____

Section five: cultural heritage

17. How often do you attend activities that are part of your African cultural heritage? (eg. Catch up with your own people or attend events with your community)
    A. every week or more often
    B. almost every week
    C. once or twice a month
    D. a few times a year
    E. less than that
    F. I don’t attend at all

If you attend these activities, what did you do during these sessions? _______________
____

18. How important is for you to pass on your cultural heritage or the traditions of your ancestors to the next generation?
    A. Very important
    B. Somewhat important
    C. Neutral
    D. Somewhat unimportant
    E. Unimportant

Section six: socio-demographic information

19. What is your age:
    A. 18-24 years
    B. 25-34 years
    C. 35-44 years
    D. 45-54 years
    E. 55-64 years
    F. 65-74
    G. 75 or above
20. What is your country of origin?________________________________
21. At what age did you came to Australia? ______________________
22. Marital status:
    A. Married
    B. Single
    C. Cohabiting
    D. Widowed
    E. Divorced
23. Have you ever been pregnant?
    A. Yes
    B. No

    If yes, how many times have you been pregnant? ____________
24. Do you have children?
    A. Yes
    B. No

    If yes, how many children do you have now and how old are they? ______________________
    ____

25. What is your approximate average household income?
    A. $0-$24,999
    B. $25,000-$49,999
    C. $50,000-$74,999
    D. $75,000-$99,999
    E. $100,000-$124,999
    F. $125,000-$149,999
    G. $150,000-$174,999
    H. $175,000-$199,999
    I. $200,000 and up
26. Would you say your household income is enough to manage the living cost of the family
    A. Difficult to manage on available income
    B. Sometimes difficult
    C. Usually manageable
    D. Easy to manage
27. What is the highest level of education you have achieved?
    A. Less than high school
    B. High school graduate
    C. Certificate or diploma level
    D. University degree
    E. Postgraduate degree
    F. Other (please specify) ______________________________________
28. What is your current employment status?
    A. Employed full-time
    B. Employed part-time
    C. Self-employed
    D. Unemployed
    E. Other: Specify______________

29. What is your religious affiliation?
    a. African traditional religion
    b. Christian
    c. Catholic
    d. Jewish
    e. Muslim
    f. Buddhist
    g. Hinduism
    h. Others (please specify) _______________
30. Through which visa pathway did you come to Australia?
    a. Family/partner
    b. Humanitarian/Refugee
    c. Student
    d. Skilled/Work
    e. Other (please specify) ____
31. How long you stayed in Australia?
    a. Less than 1 year
    b. 1-5 years
    c. 6-10 years
    d. More than 10 years
32. What is your current status in Australia?
    a. Citizen
    b. Permanent resident
    c. Temporary resident/student/visitor
    d. Humanitarian/refugee/asylum seeker

33. How do you rate your English language proficiency? Consider your writing, speaking, listening and reading abilities in English.
    a. Fluent
    b. Excellent
    c. Good
    d. Limited
    e. Very limited

End of survey.

You are invited for an interview!

We think you would be perfect for participation in individual interviews using phone, online or face-to-face with the researcher to share your experience and thoughts about traditional or complementary medicine.

You will receive a $50 voucher to reimburse you for your time or travel for the interview. Importantly, your contribution and ideas will help inform mainstream health practice and training as well as future policy. Your thoughts will help to design and improve culturally competent women's health services for migrant African women in Australia.

If you are interested in participating in the interview, please provide your contact details below and we will get in touch soon!

First Name:____
Family Name:____
Email:____
Mobile:___________________________________
