# Supplementary material for: Use of traditional and complementary medicine for maternal health and wellbeing by African migrant women in Australia: a mixed method study
Source: BMC Complement Med Ther. 2020 Feb 18;20:60. doi: 10.1186/s12906-020-2852-6 (PMC7076811; doi:10.1186/s12906-020-2852-6)
Supplement: Supplementary file 2 — Additional file 2. Interview guide. [file 12906_2020_2852_MOESM2_ESM.pdf]

Supplementary file 2: Interview guide

Use of Traditional and Complementary Medicine for Maternal Health Care among African Migrant Women in Australia

Opening/warm-up questions

- What can you tell me about adapting a new health care system in Australia?

Main questions

- The meaning of traditional/complementary for you/your community/in your culture
  - What is the first thing that comes to your mind when you think about traditional/complementary medicine
  - How are these practices/practitioners valued by yourself and the African community?
  - What role did traditional/complementary medicine have for you in Africa?
  - What role did traditional/complementary medicine have for you in Australia?
  - Have your views changed about traditional or complementary medicine since you came to Australia?
- Personal experience and attitudes on use of traditional/complementary medicine for maternal health purposes
  - What is your experience of using traditional/complementary medicine in Africa and Australia?
  - What is the main reason to use traditional/complementary medicine during pregnancy/birth or postpartum or to prepare for pregnancy?
  - In your opinion, how do you think about the safety and effectiveness of traditional or complementary medicine?
  - Who you feel more comfortable with to discuss your health issues, traditional healers or western medicine practitioners or both? Why?
- Barriers in accessing the mainstream maternity care and complementary therapy in Australia
  - Probe the discussion in terms of acculturative stress to new health care services, cultural appropriateness of women's health services, perceived prejudice and/or discrimination in getting the mainstream maternity health service
  - Probe for specific barriers in accessing and using traditional or complementary medicine
  - Are you comfortable to discuss with your doctor/midwife about your use of traditional or complementary medicine?

Ending questions

Do you have questions or comments?
